# Antiurolithiatic Activity of Extract and Oleanolic Acid Isolated from the Roots of *Lantana camara* on Zinc Disc Implantation Induced Urolithiasis

**DOI:** 10.1155/2013/951795

**Published:** 2013-05-15

**Authors:** Narendra Vyas, Ameeta Argal

**Affiliations:** ^1^Institute of Pharmaceutical Science & Research Center, Bhagwant University, Ajmer, Rajasthan 305004, India; ^2^Rajeev Gandhi College of Pharmacy, Village Salaiya, Via Danish Kunj, Kolar Road, Bhopal, Madhya Pradesh 462042, India

## Abstract

The present study was done to evaluate the antiurolithiatic activity of ethanolic extract of roots (ELC 200 mg/kg) and oleanolic acid (OA 60 mg/kg, O.A. 80 mg/kg, O.A. 100 mg/kg) isolated from roots of *Lantana camara* in albino wistar male rats using zinc disc implantation induced urolithiatic model. The group in which only zinc disc was implanted without any treatment showed increase in calcium output (23  ± 2.7 mg/dL). Cystone receiving animals showed significant protection from such change (*P* < 0.01). Treatment with OA and ELC significantly reduced the calcium output at a dose of OA 60 mg/kg (*P* < 0.01), OA 80 mg/kg (*P* < 0.01), ELC 200 mg/kg (*P* < 0.01), and OA 100 mg/kg (*P* < 0.001), as compared with zinc disc implanted group. The average weight of zinc discs along with the deposited crystals in the only disc implanted group was found to be 111 ± 8.6 mg. Group that received Cystone 500 mg/kg showed significant reduction in the depositions (*P* < 0.001). Similarly, the rats which received OA and ELC showed reduced formation of depositions around the zinc disc (*P* < 0.001). The X-ray images of rats also showed significant effect of OA and ELC on urolitiasis. Thus, OA and ELC showed promising antiurolithiatic activity in dose dependant manner.

## 1. Introduction

Urolithiasis denotes stones originating anywhere in the urinary tract, including the kidneys and bladder. However, the pathophysiologic bases for the formation of kidney and bladder stones are entirely different. Kidney stones form as a result of physicochemical or genetic derangements leading to supersaturation of the urine with stone-forming salts or, less commonly, from recurrent urinary tract infection with urease producing bacteria. Stasis in the upper urinary tract due to local anatomic anomalies may also promote or enhance stone formation in susceptible individuals. In contrast, bladder stones form almost exclusively as a result of urinary stasis and/or recurrent infection due to bladder outlet obstruction or neurogenic bladder [1].

It is estimated that about 12% of men and 55% of women have at least one episode of kidney stone during their life time. Once kidney stone develops, the recurrence rate is estimated to be 14% at 1 year, 35% at 5 years, and 52% at 10 years. The incident in general population is about 1 in 1000 adults per year. The cause of urolithiasis is still unknown but probably positive family history, overweight, obesity, or increased BMI. Some other causes include low urine volume <1500 mL/day, high dietary animal protein intake, increased urine excretion of calcium oxalate, uric acid and cystine. Urinary tract structural abnormalities leading to stasis of urine flow [2].

*Lantana camara (L. camara*), also known as Spanish Flag or West Indian Lantana, is a species of flowering plant in the verbena family, Verbenaceae, which is native to the American tropics. Its Ayurvedic names are Chaturaanga and Vanachchhedi and in Hindi it is commonly known as Raimuniya. *L. camara* has covered large areas in India, Australia, and much of Africa [3].

The present study was done to evaluate the antiurolithiatic activity of extract and oleanolic acid isolated from the roots of *L. camara* in albino wistar male rats using zinc disc implantation induced urolithiatic model.

## 2. Materials and Methods

### 2.1. Plant Material

The roots of *Lantana camara* were procured from local areas of Bhopal (Madhya Pradesh, India) and authenticated from Department of Botany, Safia College Bhopal (Voucher no. 280/bot/saf/11). The roots were then allowed to dry in air and crushed in small pieces and powdered for extraction. 

### 2.2. Plant Extraction

The powdered roots of *L. camara* were extracted with ethanol using maceration method. The extract was then dried and stored. Phytochemical screening of the extract was done and results show the presence of tannins, protein, reducing sugars, triterpenoids, and so forth. in ethanolic extract of *L. camara* roots [4].

### 2.3. Isolation of Oleanolic Acid (OA)

The powdered crude drug was defatted thrice in cold overnight with petroleum ether and then extracted exhaustively with ethanol four times over night at room temperature. The solvent was removed under vacuum at 40°C and the crude extract was dissolved in chloroform and left over night for precipitation. The precipitate so obtained was crystallized with Methanol. Precipitation and crystallization processes were repeated 4 times, which gave oleanolic acid crystals [5].

### 2.4. Animals

Healthy male albino wistar rats of 150–250 g body weight were used for this study. The animals were housed in polypropylene cages and maintained under standard conditions (12 hrs light and dark cycles, at 25 ± 30°C and 35–60% humidity). Standard palletized feed and tap water were provided ad libitum. The study was approved by Institutional Animal Ethical Committee of Sapience Bio-analytical Research Laboratory (SBRL), Bhopal, India, registered under CPCSEA, India (Registration no. 1413/a/11/CPCSEA).

### 2.5. Zinc Disc Implantation Induced Urolithiasis

#### 2.5.1. Preparation of Zinc Disc

Zinc disc: before day of implantation 36 discs were prepared having weight 20 ± 2 mg.

#### 2.5.2. Animals

Forty-two albino wistar male rats were used for the study, each weighing between 150 and 200 g. 

#### 2.5.3. Preparation of Cystone Solution

The Cystone tablets were crushed and 5 g of powder of Cystone tablet was dissolved in 100 mL 0.5% CMC solution.

### 2.6. Procedure

The wistar rats were divided into different treatment groups as sham operated group (the same surgical procedure but without implantation of zinc disc), zinc disc implanted group, zinc disc implanted group with 7 days treatment with Cystone 500 mg/kg/day [6], zinc disc implanted groups with 7 days oleanolic acid treatment at three successive doses, that is, 60 mg/kg/day, 80 mg/kg/day, and 100 mg/kg/day, and zinc disc implanted group with 7 days treatment with ELC (Ethanolic extract of *Lantana camara* roots at a dose of 200 mg/kg) dissolved in 0.5% of carboxy methyl cellulose (CMC). The groups had 6 rats in each. 

Zinc disc implantations in urinary bladders were carried out by an earlier reported method by Vermeulen et al. [7]. Prior to surgery, rats were fasted for 8–10 hours. Just before anesthesia, they were orally administered with 4 mL of water to dilate their urinary bladders. The rats were operated in sterile conditions under Ketamine (80 mg/kg/i.p.) and diazepam (4 mg/kg/i.m.) anesthesia. The urinary bladders were exposed through a suprapubic incision and a small cut was taken to open the lumen of the bladder. In zinc disc implanted groups, discs of average weight of 20 ± 2 mg were inserted and the incision was closed by 1 or 2 stitches of absorbable sterile surgical sutures (Centennial CNW 2670 6-0 USP). The abdominal incisions were sutured with sterile silk suture. The rats in sham operated groups were similarly operated except implantation of zinc disc in the bladder [8]. All the operated rats were treated topically with antibiotic dusting powder and allowed to recover for three days without any interventions. Treatment was started after the three days of recovery [9].

### 2.7. Urine Sample Collection

At the end of the treatment period, individual animal was placed in the metabolic cage for collection of 24-hour urine samples. Initially a drop of chlorhexidine gluconate was added to the test tubes as a preservative.

### 2.8. Radio Graphical Examination

Radio graphical examination was done before sacrificing the animals to confirm the formation of stones. The animals were kept under light ether anaesthesia in anteroposterior position on the X-ray board to expose the pelvic region, with digital X-ray instrument (GE-525 DX, USA) by Fuji computerized radiographic system Japan. The film focus distance was 60 inches and machine was operated at 43 kV peak, 2 mA [10].

### 2.9. Weight of Stone

The weight of urinary calculi determined by sacrificing the animals at the end of study. The urinary bladders were exposed and zinc disc along with the adhered crystals was removed and wrapped in separated polyethylene bags.

## 3. Results

In antiurolithiatic activity study, zinc discs were implanted in the urinary bladder of rats. The zinc discs initiate and fasten the nucleation process which is rate limiting step in the formation of urinary calculi. The urine volume was decreased in sham control (Incision treated) (2.1 ± 0.2) as well as in zinc disc implanted group (1.6 ± 0.1) as compared to naïve control group (Table 1), suggesting the obstruction of urine excretion due to nidus formation, crystal aggregation, oxalate salt saturations, and crystal retention within urinary bladder. 

Rats treated with OA showed dose dependent increase in urine output but the values were not statically significant.

Urolithiatic animals showed increase in calcium output through urine. In zinc disc implantation model, the group in which only zinc disc was implanted without any treatment showed increase in calcium output at the 10th day after implantation (23 ± 2.7 mg/dL). Cystone receiving animals showed significant protection from such change (*P* < 0.01). Treatment with OA and ELC significantly reduced the calcium output at a dose of OA 60 mg/kg (*P* < 0.01), OA 80 mg/kg (*P* < 0.01), ELC 200 mg/kg (*P* < 0.01), and OA 100 mg/kg (*P* < 0.001), as compared with zinc disc implanted group (Table 1).

At the end of 10 days, the rats were sacrificed; the zinc discs were removed and weighed. The average weight of zinc discs along with the deposited crystals in the only disc implanted group without any treatment was found to be 111 ± 8.6 mg. Group that received Cystone 500 mg/kg, showed significant reduction in the depositions hence there was significant difference in the weight of bladder content as compared to zinc disc implanted group (*P* < 0.001). Similarly, the rats which received OA 60 mg/kg/day, 80 mg/kg/day 100 mg/kg/day, and ELC 200 mg/kg/day showed reduced formation of depositions around the zinc disc (*P* < 0.001) (Table 1).

**Figure 1:**
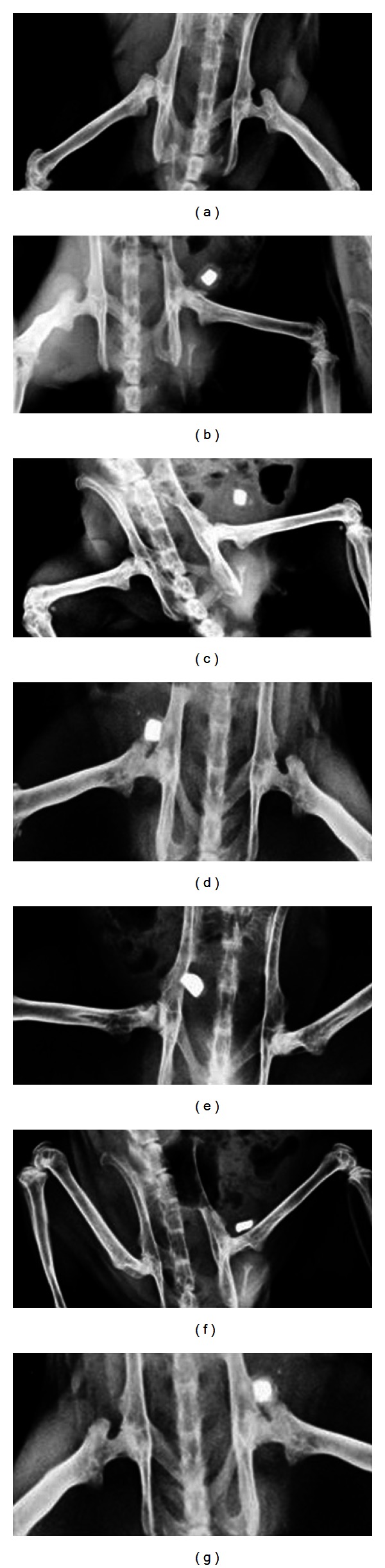
X-ray of image of rats in zinc disc implantation model of urolithiasis. (a) Control, (b) zinc disc implanted, (c) Cystone treated, (d) OA 60 mg/kg, (e) OA 80 mg/kg, (f) OA 100 mg/kg, and (g) ELC 200 mg/kg.

Figure 1 shows the results of effects of OA and ELC on zinc disc implantation model of urolithiasis.

As shown in Figure 1, the X-ray images of rats revealed presence of deposits around the implanted zinc disc within 10 days after implantation. Treatment with Cystone at dose of 500 mg/kg/day significantly reduced formation of such deposits within 10 days.

The effects of OA 60 mg/kg/day, 80 mg/kg/day, 100 mg/kg/day, and ELC 200 mg/kg/day reduced the formation of deposits around implanted disc. These observations were confirmed in actual weights of the bladder contents. 

## 4. Discussion and Conclusion

Various animal models have been employed in the previous research to induce urolithiasis including several crystal- inducing drugs (CID), such as ethylene glycol, oxalate, gentamicin sulfate, glycolic acid, ammonium chloride, and L-hydroxyproline. However, most of these models were associated with nephrotoxicity [11]. However, zinc disc model induced urinary calculi without severe renal damage and this animal model is always used to mimic the etiology of urinary stone formation in humans. Zinc disc implantation model is primary model in which the main component of formed crystals is magnesium ammonium phosphate [12]. 

Urinary stone formation is based on supersaturation of urinary salts and crystal retention in the urinary tract. Hyperoxaluria and hypercalciuria are important risk factors in the pathogenesis of urinary stone formation. 

In zinc disc implantation model, the urine output was decreased in disc implanted group as compared to naïve control group. This suggests obstruction of urinary bladder due to formation of large urinary calculi. Partial obstruction of the urinary bladder outlet leads to a compensatory growth of the detrusor smooth muscle cells, and this occurs as a feedback response to the increased intravesical pressure required to empty the bladder. Partial obstruction of the urinary bladder was also shown to induce a decrease in the density of autonomic innervation and sensitivity to the muscarinic agonist. In literature survey, OA was found to increase the secretion of insulin by pancreatic *β*-cells either by stimulating M3 in pancreatic *β* cells or through the released acetylcholine from cholinergic nerve terminals. In fact, increase of diuresis could reduce supersaturation of the urine with precipitating substances which is normally associated with formation of urinary calculi [13]. The increase in urine output by OA could be via activation muscarinic receptor in the bladder muscles along with other mechanisms. In ELC urine output was also increased. 

On the 10th day after implantation, the urine calcium was increased in disc implanted animals; this is in accordance with previous study reported by [14]. Urinary calcium is a factor that favors the process of nucleation and precipitation of calcium oxalate or apatite (calcium phosphate) from urine and subsequent crystal growth. Treatments with Cystone significantly reduced the increased calcium output. OA and ELC also reduced such increased calcium output in dose dependent manner. Treatment with OA and ELC was found to inhibit the formation calculi which are mainly composed of magnesium ammonium phosphate. Treatment with Cystone (500 mg/kg) also has similar effects. Cystone has already been proved by Mitra et al. (1998) [6] to possess antiurolithiatic action; in our study we found that oleanolic acid has potent antiurolithiatic action at comparatively very low dose range as compared to Cystone. 

The formation of calculus in urinary bladder was also confirmed by X-ray radiological investigation. Drugs treatment shows only zinc disc in radiographic studies. Digital X-ray radiography provides a means to easily monitor the crystal growth. As evident in this study, within 10 days after implantation, depositions could be visualized around the implants. Hence, use of this technique can help the investigator to determine the time at which study is to be terminated. 

Thus our data indicates that while evaluating an antiurolithiatic drug by zinc disc implantation model, X-ray radiography should be used to monitor the growth of deposit around the implants and as soon as sufficient deposition is revealed in control groups, the study should be terminated so as to minimize undue stress to the animals. Recently, instead of zinc disc other foreign bodies which can be easily processed for evaluation have been suggested to be implanted in the bladder.

## Figures and Tables

**Table 1:** Effect of oleanolic acid and ELC on zinc implantation model.

Serial number	Groups	Urine output (mL)	Urinary calcium output (mg/dL)	Weight of calculi (mg)
(1)	Control	2.6 ± 0.2	—	—
(2)	Sham Control	2.1 ± 0.2	10 ± 3.1**	—
(3)	Disc Implanted	1.6 ± 0.1*	23 ± 2.7	111 ± 8.6
(4)	Cystone 500 mg/kg	2.6 ± 0.3	12 ± 2.7**	6.7 ± 2.9***
(5)	OA 60 mg/kg	1.9 ± 0.1	12 ± 0.7**	11 ± 2.4***
(6)	OA 80 mg/kg	2 ± 0.2	10 ± 2**	11 ± 1.9***
(7)	OA 100 mg/kg	2.7 ± 0.3	8.4 ± 1.8**	4.1 ± 1.3***
(8)	ELC 200 mg/kg	2.4 ± 05	11 ± 1.2***	13 ± 1.7***

Value represents; mean ± SEM. Statistical analysis was performed by Dunnett's multiple comparison test. *P < 0.05, as compared with naive control, **P < 0.01, and ***P < 0.001 as compared with disc implanted group.
